# Evaluation of energy spectrum CT for the measurement of thyroid iodine content

**DOI:** 10.1186/s12880-016-0151-y

**Published:** 2016-08-12

**Authors:** Weiguang Shao, Jingang Liu, Dianmei Liu

**Affiliations:** Department of Imaging Center, the Affiliated Hospital of Weifang Medical University, Weifang, 261031 China

**Keywords:** Energy spectrum CT imaging, Thyroid, Iodine content

## Abstract

**Background:**

This study aims to provide a reference for the diagnosis of iodine deficiency disorder by evaluating the normal thyroid iodine content by energy spectrum computed tomography (CT) and calculating the iodine content ratio of thyroid to sternocleidomastoid.

**Methods:**

The thyroid glands of 226 patients were scanned by energy spectrum CT, and the images were analyzed using the GSI Viewer software. Based on the imaging findings, the iodine levels of the thyroid lobes as well as the bilateral sternocleidomastoids were evaluated, and their iodine content ratios were calculated.

**Results:**

No statistically significant difference was found in the thyroid iodine content between the left and right thyroid lobes (*p* > 0.05). However, there was a significant difference in the thyroid iodine content between men and women (*p* < 0.01). Additionally, the thyroid iodine content was found to decrease gradually with age. The iodine content ratio of thyroid to sternocleidomastoid was 96.6271 ± 33.2442.

**Conclusion:**

Gemstone energy spectrum CT can be used for the measurement of thyroid iodine content in the human body. It can play a significant role in the diagnosis of iodine deficiency disorder.

## Background

Iodine, an essential trace element for the human body, is closely related to the thyroid and is essential for the synthesis of the thyroid hormones. Iodine deficiency or excess iodine can cause thyroid dysfunction [1, 2], leading to abnormal iodine content in the thyroid tissues, which results in disorders of the thyroid and other organs of the body [3–5]. The iodine intake levels of the thyroid and *in vivo* storage concentrations can be evaluated by measurement of the iodine content of the thyroid tissue. These values can be used to determine whether the thyroid dysfunction is caused by iodine deficiency or excess, which is clinically significant in the diagnosis of thyroid diseases [6]. Previously, the iodine content of the body was indirectly determined by the measurement of urine iodine levels [7, 8] and thyroid iodine absorption rates [9]. However, the iodine content of the thyroid glands cannot be measured by these methods. Thyroid iodine content can be determined by the conversion of thyroid CT values; however, X-rays, which are used to achieve excitation in this process, have a limited spectral energy range, which inevitably leads to inaccuracies in the CT values, thus affecting the results of quantitative diagnosis [10, 11]. Gemstone energy spectrum CT is based on the differences in the X-ray attenuation coefficients of different materials. This technique can be used to obtain not only monoergic images, but also substance-separation images [12, 13]. Iodine-based substance separation images are very sensitive to iodine deposition and exhibit good resolution of tissues such as the thyroid gland; they can, therefore, be used for the quantification of the thyroid iodine content. In the present study, the possibility of energy spectrum CT iodine-based substance-separation imaging completely or partly replacing the previous methods for the measurement of thyroid iodine content was evaluated.

## Methods

### Subjects

Patients who underwent cervical and thyroid CT imaging between October 2010 and May 2011 were enrolled in this study. All of the patients had undergone energy spectrum CT imaging of the thyroid and sternocleidomastoid. None of the patients had received thyroid preparations, iodine products, or special foods such as laver, kelp, or seaweed. Patients with cysts, adenoma, calcifications, inflammation, and other disorders and/or dysfunctions of the thyroid were excluded. A total of 226 subjects between the ages of 18 and 77 years (mean age, 46 ± 17 years), including 119 male and 107 female patients were enrolled. This study was approved by the ethics committee of an affiliated hospital of the Weifang Medical College, and informed written consent had been obtained from all of the patients.

### Computed tomography scanning and post-processing

Energy spectrum CT scanning was performed using the Discovery CT750 high definition (HD) scanner (GE Healthcare, Milwaukee, WI, USA), with the following scanning parameters: section thickness, 5.0 mm, with 5.0-mm intervals; thread interval, 0.984:1; speed, 39.37 mm/rotation; and rotation time, 0.8 s. The imaging data were reconstructed at a reconstruction thickness and interval of 0.625 mm and transmitted to AW4.4 workstations, where they were analyzed using the GSI viewer software. Monoergic images with optimal thyroid contrast-to-noise ratios (CNRs) were obtained from the energy spectrum curve. On the iodine-based substance-separation images, the largest thyroid layer was selected for the measurement of iodine content in the right and left thyroid lobes within 50 mm^2^ circular areas. The iodine content was measured at two or three points on the upper pole of each lobe, and the mean value of these measurements was calculated. The iodine content in the bilateral sternocleidomastoids was also measured, and the iodine content ratio of thyroid to sternocleidomastoid was determined.

### Statistical analysis

The data were recorded as the mean values ± standard deviations (SD) and analyzed using the SSPS v13.0 (Chicago IL, USA) software. Comparison of the thyroid iodine content between the male and female patients was performed using the *t*-test, while comparison of the thyroid iodine content among different age groups was performed by analysis of variance (F-test). Pairwise comparisons between the groups were performed using the SNK q-test. The level of significance of the test was determined at *α* = 0.05, and statistical significance was determined at *p* < 0.05.

## Results

### Thyroid iodine content in the right and left lobes

A total of 226 right and 225 left thyroid lobes (because of one patient with an absent left lobe) were evaluated. The mean iodine content in the left lobe was 1.5230 ± 0.4271 mg/cm^3^ and that in the right lobe was 1.5236 ± 0.4365 mg/cm^3^ (Table 1). The difference in iodine content between the right and left lobes was not statistically significant (*t* = 0.0084; *p* > 0.05).

**Table 1:** Comparisons of the thyroid iodine content between the right and left lobes

	No. of cases	Iodine content (mg/cc)	*t* value	*P* value
Left lobe	225	1.5230 ± 0.4271	0.0084	0.9933
Right lobe	226	1.5236 ± 0.4365		

Data were shown as mean ± SD

### Thyroid iodine content in male and female patients and iodine content ratio of thyroid to sternocleidomastoid

**Fig. 1:**
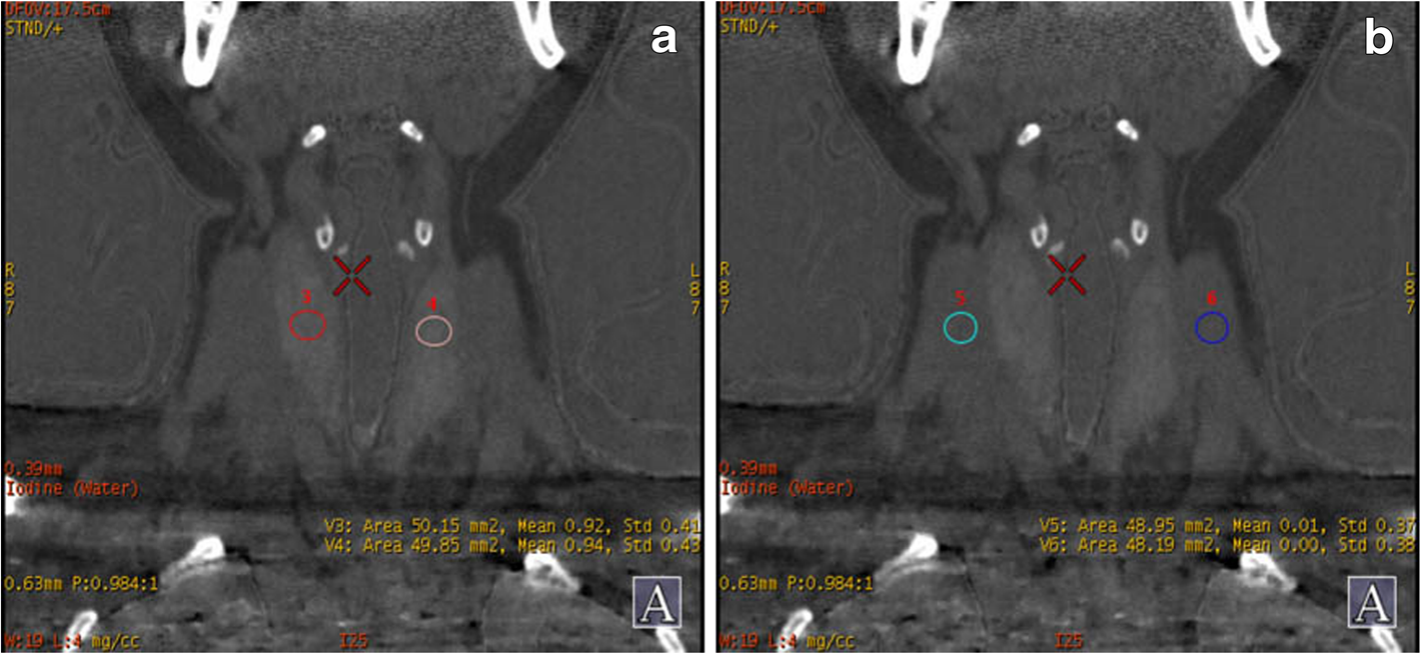
**a** Was iodine-based image for determining the iodine content in thyroid glands. **b** was iodine-based image for determining the iodine content in bilateral sternocleidomastoids

The mean value of the total iodine content in the thyroid glands was 1.5233 ± 0.4318 mg/cm^3^. The difference in iodine content between the male and female patients was statistically significant (*t* = 3.4743; *p* < 0.01; Fig. 1 and Table 2). The iodine content ratio of thyroid to sternocleidomastoid (0.0161 ± 0.0615 mg/cm^3^) was 96.6271 ± 33.2442, and it showed no statistically significant differences between the male and female patients (*t* = 0.3817; *p* > 0.3817; Fig. 1 and Table 2).

**Table 2:** Iodine content in thyroid and sternocleidomastoid of men and women

Groups	No. of cases	Iodine content (mg/cm^3^)		Iodine ratio of thyroid to sternocleidomastoid
		Thyroid glands	Sternocleidomastoid	
Men	119	1.6395 ± 0.4105*	0.0175 ± 0.0635	94.6250 ± 37.3621
Women	107	1.4238 ± 0.3832	0.0145 ± 0.0613	98.0000 ± 29.0737

Data were shown as mean ± SD. *P < 0.01, compared with women

### Optimal CNR of the thyroid glands

**Fig. 2:**
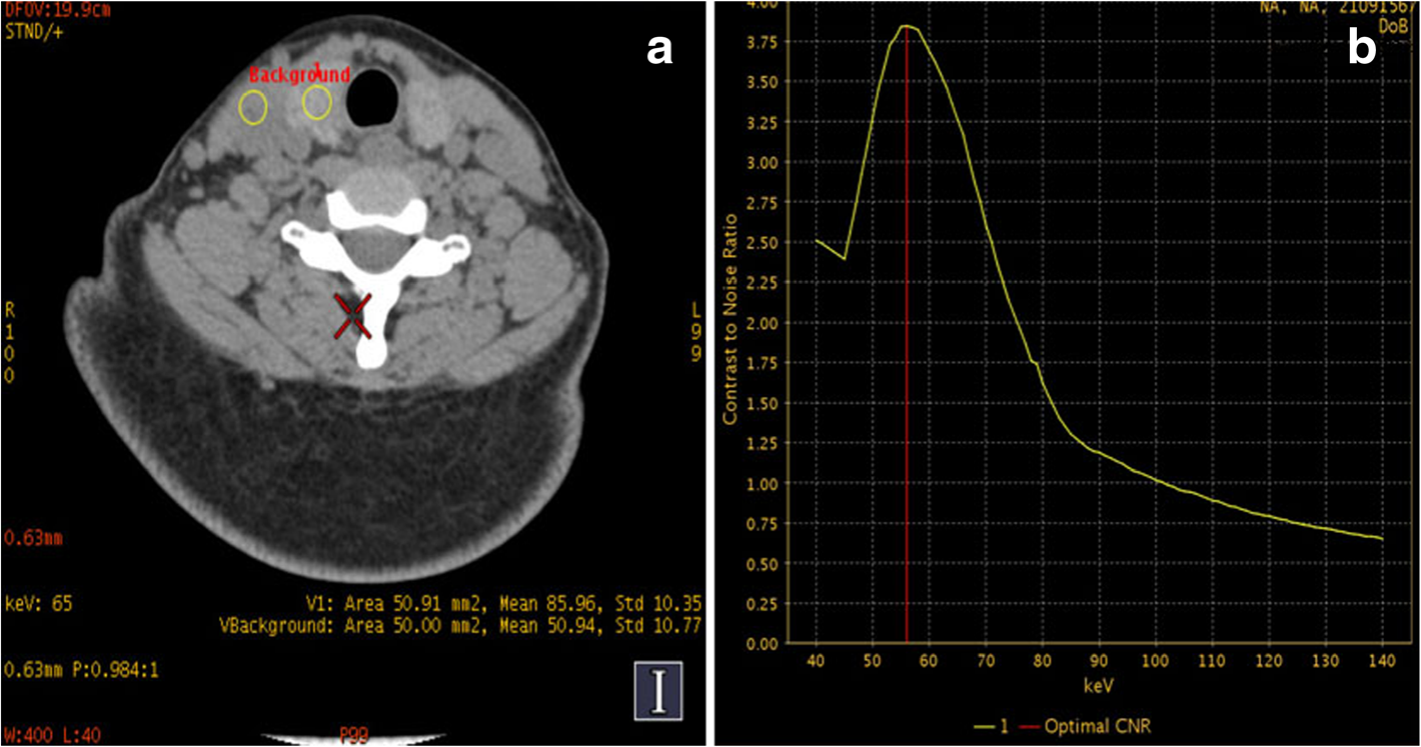
**a**. Two ROIs were drawn in right thyroid gland and ipsilateral sternocleidomastoid respectively. **b**. Using the ipsilateral sternocleidomastoid as a contrast, the right thyroid gland had the best contrast to noise ratio (57 kev) to the surrounding tissues

Monoergic images with optimal CNR of the thyroid glands against the sternocleidomastoid were achieved at 57.0167 ± 2.7647 keV (Fig. 2).

### Thyroid iodine content in different age groups

The thyroid iodine content showed significant differences among the different age groups evaluated in the present study (*F* = 9.66; *p* < 0.01). Patients below 40 years of age had markedly higher thyroid iodine content than patients between the ages of 40 and 60 years and those above 60 years of age (*q* = 5.6195 and 5.6195, respectively; *p* < 0.01, both; Table 3). However, the difference in thyroid iodine content between patients between the ages of 40 and 60 years and those above 60 years of age was not statistically significant (*q* = 0.3166; *p* > 0.3166; Table 3).

**Table 3:** Comparisons of thyroid iodine content among different age groups

Age groups	No. of cases	Iodine content (mg/cm^3^)		*q* value	*P* value
<40 years (a)	59	1.7256 ± 0.4631	a *vs.* b	5.6195	<0.01
40 to 60 years (b)	96	1.4517 ± 0.3643	a *vs.* c	5.4158	<0.01
>60 years (c)	71	1.4368 ± 0.3465	b *vs.* c	0.3166	>0.05

## Discussion

### Characteristics of energy spectrum CT

Energy spectrum imaging was first studied in the 1970s [14]. This was followed by the clinical application of this research in dual energy imaging [15]. In the 2000s, it was almost possible to achieve substance separation with the dual-energy subtraction technique by means of CT scanning [16]. Using the instantaneous double kVp technique, it was possible to acquire a series of monoergic images at specific energy levels by means of energy spectrum CT in order to effectively avoid the beam hardening effect and improve the CNR of the images, thus obtaining stable and precise CT values. These advances resulted in the replacement of conventional CT, which is based on the variation of a single parameter, as the diagnostic modality of choice with energy and multi-parameter-based CT evaluation [17]. Image acquisition by CT is based on the attenuation of X-rays in the objects of interest. Each material has its own characteristic curve correlating the changes of the mass absorption coefficient with energy. Because of the inherent differences in their energy attenuation coefficients, different materials in an object can be identified and quantified by excitation at two different energy levels. This is the physical basis of substance-separation imaging [18]. By means of substance-separation imaging, each structure can be broken down into separate substances with different X-ray attenuation coefficients, following which, contrast images of these substances can be acquired and used for the qualitative analysis of the contents. Since iodine-based substance-separation imaging exhibits high sensitivity in the imaging of iodine deposits, it aids in better visualization of iodine-rich tissues such as the thyroid glands. Therefore, iodine-based thyroid density images can be used for the quantitative analysis of the iodine content of the thyroid glands.

### Limitations of the previous evaluation methods

Thyroid iodine content accounts for about 20–50 % of the total iodine content of the human body. It has been reported that excessive iodine intake as well as its deficiency can lead to thyroid dysfunction, which exhibits a U-shaped relationship with iodine intake [19, 20]. Measurement of iodine content can reveal the amount of iodine reserve *in vivo* as well as the recent iodine intake levels. Previous determination methods mostly focused on the measurement of either urine iodine [7, 8] or the rate of thyroid iodine absorption [9]. However, the results obtained by such methods can be affected by the iodine content contributed by food and renal and gastrointestinal functions. The iodine content of the thyroid can also be analyzed by measurement of the CT values using a specific formula; however, the spectral range of X-rays is limited. Moreover, conventional CT image acquisition cannot effectively avoid the beam hardening effect in order to obtain stable and accurate CT values; this leads to inaccuracies in quantitative analysis [10, 11].

### Advantages and clinical value of energy spectrum CT for the measurement of thyroid iodine content

Gemstone energy spectrum CT can be used to effectively determine the iodine content of the thyroid using iodine-based substance-separation images transformed from the X-ray attenuation curve. Errors due to inaccuracies of the CT values can be avoided, and accurate measurements of the iodine levels in the body can be obtained. Based on the results of specimen imaging studies by Li et al. [21], dual-energy CT can be considered as a promising quantitative approach for the differentiation of malignant and benign thyroid nodules. In the present study, the results of analysis of normal thyroid glands revealed no statistically significant differences in iodine content between the left and right thyroid lobes; however, significant differences in thyroid iodine content were observed between the male and female subjects, which might be associated with the differences in endocrine hormonal levels between the two sexes. We also found a gradual decline in the thyroid iodine content with increasing age, which suggests that thyroid function, including iodine reserve and uptake, exhibits a tendency to decline with age. The results of comparison among the different age groups revealed no significant differences in thyroid iodine content between patients between the ages of 40 and 60 years and those above 60 years of age. However, the thyroid iodine content of the patients of both groups was lower compared to that of the patients below 40 years of age, which indicates that thyroid function might start to decline from the age of 40. However, the relationship between age and thyroid function needs verification by further studies with larger sample sizes.

The iodine content ratio of thyroid to other tissues, which is normally about 100:1, is an important index in the diagnosis of iodine deficiency disorders; this ratio has been reported to be high as 400:1 in patients with iodine deficiency disorders [5]. In cases where the ratio is higher than 100:1, the concentration of iodine absorbed should be evaluated. In cases where the ratio is close to 400:1, the patients are considered as exhibiting serious iodine deficiency. In the present study, the iodine content ratio of thyroid to sternocleidomastoid was 96.6271 ± 33.2442. Therefore, this value can be used as the reference for the diagnosis of iodine deficiency disorders by measurement of the thyroid iodine content by energy spectrum CT. Gemstone energy spectrum CT can be used not only to accurately measure the iodine content of the thyroid glands and determine the iodine content ratio of thyroid to sternocleidomastoid, but also to determine the volume of the thyroid glands and evaluate any complications because of other thyroid diseases. Thus, energy spectrum CT is more advantageous than conventional CT in the evaluation of thyroid function and morphology.

### Limitations of this study

First, the sample size in the present study was too small, which resulted in the inclusion of subjects of a wide range of ages. Moreover, because of the small sample size, the age groups could not be evaluated in terms of sex-specific differences in thyroid iodine content. Further studies with larger sample sizes including higher numbers of subjects of each age group are required for obtaining more reliable results. Second, we positioned the ROIs only in the upper poles of the thyroid glands; however, whether the distribution of iodine is consistent across the entire thyroid gland has yet to be confirmed. Positioning of the ROIs only in the upper or lower poles of the thyroid gland might lead to inconsistency in the results. Third, the equipment used was expensive and involved the use of radioactive imaging agents. Therefore, its application in the routine screening of abnormal thyroid function might be challenging.

## Conclusion

Gemstone energy spectrum CT can be used for the evaluation of thyroid iodine content in the human body. In the present study, imaging by this method revealed a gradual decrease in thyroid iodine content with age. Therefore, gemstone energy spectrum CT is a promising tool for the diagnosis of iodine deficiency disorder.

## Abbreviations

CT, computed tomography; HD, high definition; CNR, contrast to noise ratio; SD, standard deviation.
